# Evaluation of a Standardized Protocol for Plasma Rich in Growth Factors Obtention in Cats: A Prospective Study

**DOI:** 10.3389/fvets.2022.866547

**Published:** 2022-04-14

**Authors:** Laura Miguel-Pastor, Katy Satué, Deborah Chicharro, Marta Torres-Torrillas, Ayla del Romero, Pau Peláez, José M. Carrillo, Belén Cuervo, Joaquín J. Sopena, José J. Cerón, Mónica Rubio

**Affiliations:** ^1^Bioregenerative Medicine and Applied Surgery Research Group, Department of Animal Medicine and Surgery, CEU Cardenal Herrera University, CEU Universities, Valencia, Spain; ^2^García Cugat Foundation CEU-UCH Chair of Medicine and Regenerative Surgery, CEU Cardenal Herrera University, CEU Universities, Valencia, Spain; ^3^Interdisciplinary Laboratory of Clinical Analysis, University of Murcia, Murcia, Spain

**Keywords:** cat, platelet count, Platelet-Rich Plasma, platelet-derived growth factor BB, transforming growth factor β1, PRGF®

## Abstract

**Introduction:**

Platelet-rich plasma (PRP) is an autologous plasma with platelet (PLT) concentration above that of whole blood (WB). PLTs contain growth factors (GFs) that promote tissular repair.

**Objectives:**

To determine and compare the concentrations of PLT, red blood cells (RBC) and white blood cells (WBC) between WB samples, PRP and platelet poor plasma (PPP) samples; and to analyze the concentrations of platelet-derived growth factor BB (PDGF-BB) and transforming growth factor β1 (TGF-β1) in the PRP and PPP of healthy adult cats using a standardized protocol with PRGF®-Endoret® characteristics.

**Material and Methods:**

WB was collected from 30 cats. PRP was obtained following three centrifugation protocols using PRGF®-Endoret® technology: 255, 260, and 265 g for 10 min each. The cellular components, RBC, WBC, PLT, and the concentrations of PDGF-BB and TGF-β1 in the PRP and PPP fractions were determined for each protocol.

**Results:**

PLTs in the PRP fraction were statistically higher than WB, with no statistical differences between PPP and WB. In PRP fraction, PLT concentration was increased 1.4 times on average at 255 g; 1.3 times at 260 g and, 1.5 times at 265 g without statistical differences among them. The mean platelet volume (MPV) was significantly higher in WB compared to PRP and PPP fractions without significant differences between protocols. Compared to WB, the number of RBCs and WBCs was reduced by 99% and by more than 95% in PRP and PPP respectively, without significant differences between protocols. PDGF-BB concentrations were statistically higher in PRP than in PPP fractions, however, TGF-ß1 concentrations did not vary between fractions at 260 g. Comparing the three protocols within PRP and PPP fractions, no differences in PDGF-BB and TGF-ß1 concentrations were observed.

**Clinical Relevance:**

The study shows scientific evidence regarding the obtention of PRP in cats using the PRGF®-Endoret® technology for the quantification of PDGF-BB and TGF-ß1. At 265 g for 10 min, PLT concentration was increased 1.5 times with unnoticeable erythrocytes and leukocytes in the samples. These results clearly show that the PRGF®-Endoret® methodology is suitable to obtain PRP in cats. Further studies are needed to determine the clinical efficacy of the obtained PGRF in the treatment of different pathologies in cats.

## Introduction

Platelet-Rich Plasma (PRP) is the fraction of autologous plasma that contains a platelet (PLT) concentration above baseline (1). PLT α-granules contain active substances such as chemokines, cytokines, and growth factors (GFs) among others. GFs include platelet-derived growth factor (PDGF, A-B-C), insulin-like growth factor 1 (IGF-1), transforming growth factor β1 (TGF-β1), vascular endothelial growth factor (VEGF), hepatocyte growth factor (HGF), epithelial growth factor (EGF), fibroblast growth factor (FGF) and connective tissue growth factor (CTGF). Therefore, PLTs are an important reservoir of GFs, which are biologically active polypeptides involved in chemotaxis, migration, cell proliferation and differentiation, angiogenesis, and extracellular matrix synthesis (2–7). Additionally, it has been demonstrated that PLTs are able to recruit, stimulate, and provide a scaffold for stem cells, which supports the use of GFs together with stem cells to prompt tissue regeneration and repair processes, as previously reported in dogs by this same research group (8–10). PRP therapy has its bases in the role that platelet growth factors (PGFs) play during the three phases of wound healing and repair cascade: inflammation, proliferation, and remodeling (11).

Currently, there is a wide variety of scientific evidence showing promising results on the use of autologous PRP in canine and equine species to treat some orthopedic pathologies (8, 10, 12–15). PRP and PRGF are both plasma derivates; PRP is a PLT concentrate with no red blood cells (RBC) present in it, while PRGF is a subtype of PRP characterized by a moderate PLT concentration and the absence of leukocytes (WBC). Compared to PRP, PRGF prevents the proinflammatory effects of proteases and acid hydrolases contained in WBC, reducing the side effects of these cells such as pain, and inflammation. Moreover, PRGF is a 100% autologous and biocompatible preparation elaborated by a single centrifugation process (16–19). The literature regarding the obtention and clinical use of PRP in cats is very scarce, and, no PRGF study has been reported in cats, thus further studies for standardization and validation of PRP and PRGFs protocols are necessary in this species (20–22).

There is not a standardized protocol for PRP obtention, thus, the composition of different PLT derivates vary greatly in PLT, WBC and RBC content. Moreover, the concentration of GFs and fibrin, led to a tremendous variety of PRP-like products that are described by different terminologies and abbreviations (11, 23, 24). The classification of these products is based on the type of activation, PLT and GFs concentration, and the presence or absence of WBC or fibrin (25, 26). Based on the fibrin architecture and cell content, PLT products are classified into four main families: Pure Platelet-Rich Plasma (P-PRP), such as the Plasma Rich in Growth Factors Endoret (PRGF®-Endoret®) technique; Leukocyte- and Platelet-Rich Plasma (LR-PRP), such as Biomet GPS system; Pure Platelet-Rich Fibrin (P-PRF), such as Fibrinet; Leukocyte; and Platelet-Rich Fibrin (L-PRF), such as Intra-Spin L-PRF. All these products present different biological properties and mechanisms of action, as well as different clinical indications (27). Revisions to terminology and new classification criteria are still evolving today (11).

Furthermore, the ideal PLT concentration and leukocyte types are still being discussed (11). PRGF®-Endoret® Technology from BTI Biotechnology Institute has provided precise technology to obtain this PRGF and several studies had assessed its clinical use in wound healing, tissue regeneration in oral implantology, orthopedics and sports medicine, as well as treatment of corneal ulcers, among other studies (28, 29). The main characteristics of the PRGF®-Endoret® are the use of a single centrifugation and the extremely decrease of WBC and RBC. This technology diminishes the proinflammatory activity (29). Even though the collection system is similar for all species, different centrifugation protocols have been described for each species. The differences between platelet's sizes and the tendency to pseudothombocytopenia in cats make it necessary to standardize a specific blood centrifugation protocol for PRP obtention in cats (22, 23, 30).

There is currently no commercialized PRP system for cats and PRP obtention protocols for this species is still far from being standardized and the optimal characteristics compatible with PRGF®-Endoret® suitable for clinical application in feline medicine have not been achieved (21, 22, 31, 32). In other species such as rabbits and dogs, the mean PLT enrichment of PRP Endoret® preparations was ~1.5 times higher than that of WB (33, 34). Silva et al. obtained a PLTs concentrate (PC) with significantly higher amounts of GFs compared to plasma, but RBC and WBC where present on it. That indicates that PC can be experimentally used in feline medicine (20). In addition, a study published by the Clinical Hospital of the University of Padua (Italy) showed wound healing in a cat using canine PRP (35). Moreover, two recent articles describe the use of a platelet-rich fibrin matrix in the resolution of an oronasal fistula (36) and the application of PRP in a cutaneous wound secondary to mammary gland tumor removal with satisfactory results (37). In addition, plasma-rich fibrin (PRF), a second-generation PC used to regenerate tissues and promote early healing without risk of infection, has been identified and proposed to treat different conditions in cats (38). Compared to PRP and PGRF, PRF are preparations with a high-density fibrin network. Per definition, these products only exist in a strongly activated gel form and cannot be injected or used like traditional fibrin glues. Furthermore, because of their strong fibrin matrix, they can be handled like a solid material for other applications (27). Since PRP therapy offers enormous potential as an alternative treatment for degenerative diseases and tissue regeneration, more studies are needed to evaluate the clinical efficacy of PRP use in cats (20–22, 31, 39). Even though there are some reports of the application of PRF in cats, and several surgical conditions involving skin, soft tissues, and orthopedics would also justify the use of these products (40).

We hypothesize that P-PRP can be obtained in cats using PRGF®-Endoret® technology. Therefore, the purposes of this study were (I) to determine and compare the concentrations of PLT, RBC and WBC between samples of WB and in the PRP and PPP fractions and (II) to analyze the concentrations of PDGF-BB and TGF-β1 in PRP and PPP fraction in healthy adult cats using a standardized protocol with PRGF®-Endoret® characteristics: moderately PC and to avoid WBC and RBC employing a single centrifugation.

## Materials and Methods

### Animals

This clinical study was assessed and approved by the CEU Cardenal Herrera University Committee of Ethics in Animal Research and by the relevant regional authorities (code: 2018/VSC/PEA/0196) according to the Spanish Policy for Animal Protection (RD53/2013), which complies with European Union Directive 2010/63/UE.

A prospective study was performed from March 2019 to December 2021 in the Veterinary Hospital of the Cardenal Herrera-CEU University. A total of 30 domestic and non-pedigree healthy cats were included in the study. Cats came to our hospital for a routine medical check-up, an informed consent was signed by cats' owners to participate in the study. Inclusion criteria for this study was the following: all cats were tested by the commercially available combined enzyme-linked immunosorbent assay (ELISA) kit for FIV antibody and FeLV p27 antigen (Idexx SNAP Combo FeLV Ag/FIV antibody test) providing negative results. A complete blood count and plasma biochemistry were performed for each animal, reporting results within the reference range values for the feline species. None of the animals received medical treatment during the last 6 months prior to the study. Each patient was monitored by a veterinarian during the development of the procedure. Animals receiving treatment during the previous six months or currently, presence of diseases or testing positive for FeLV or FIV were excluded from the study.

### Blood Collection

In order to ensure correct sampling, blood collection was carried out under sedation using a combination of intramuscular butorphanol (0.3 mg/kg), dexmedotomidine (12 μg/kg) and alfaxalone (0.8 mg/kg). After sedation, the cephalic vein was catheterized using a 22 G catheter to collect 0.5 ml of blood which were immediately transferred to 0.5 ml tube containing K_3_-EDTA (BD Vacutainer; Becton, Dickinson) for blood count as a WB. Thereafter, a total of 27 ml of blood were collected from the jugular vein under sterile conditions in three vacutainer sodium citrate 3.8% tubes (Blood-Collecting Tubes®, BTI Biotechnology Institute, Alava, Spain), 9 ml in each tube, and subsequent analysis of GFs was carried out. Less than 10% of the total blood volume was collected in each patient and after blood collection, every cat received 27 ml of acetated Ringer's solution IV during the first 30 min to restore the vascular volume and to prevent complications such as hypotension or hypovolemia.

### Preparation of Platelet Rich Plasma

**Figure 1:**
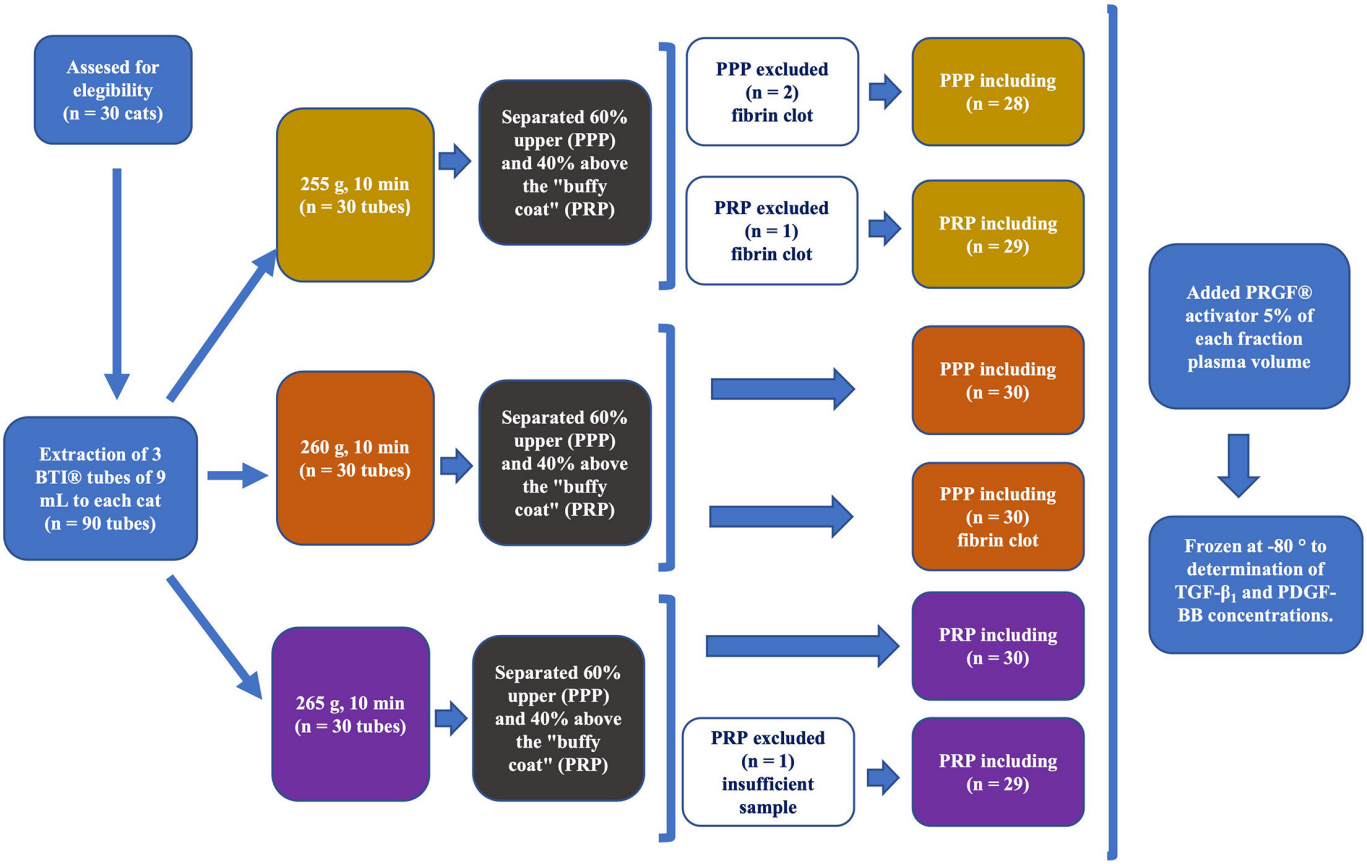
Schematization of the study design: blood sample obtention, centrifugation following the three protocols using the PRGF®-Endoret® methodology, PRP and PPP fractions obtention, subsequent activation of both fractions with BTI-activator and freezing at −80°C for subsequent GFs analysis.

Figure 1 shows the procedure for PRP obtention using the PRGF®-Endoret® methodology followed in the study. The PRP was prepared according to the PRGF®-Endoret® methodology (41–43). Samples collected in sodium citrate tubes were immediately centrifugated at room temperature in a PRGF®-Endoret® System IV centrifuge (BTI Biotechnology Institute S.L.) following three different centrifugation protocols: 255 g x 10 min, 260 g x 10 min and 265 g x 10 min. After blood centrifugation three layers are obtained: RBCs, the “buffy coat,” and the plasma. The plasma was separated into two fractions: the PPP fraction was the upper 60% and was pipetted, and the PRP fraction, equivalent to the 40% of the plasma, is located just above the “buffy coat” and was also pipetted. The plasma pipetting and blood collection procedure were carried out under maximum sterile conditions with a laminar flow hood and always by the same researcher. The samples were transferred to individual fractionation tubes with no additive (PRGF® fractionation tubes, BTI, Biotechnology Institute, Álava, Spain). Subsequently, PRP and PPP were activated by adding calcium gluconate 10% (PRGF® activator, BioTechnology Institute, Álava, Spain) (5% of the plasma volume) to achieve PLT degranulation and GFs release obtaining PRGF. The samples obtained were aliquoted into eppendorf tubes and immediately after activation were frozen at −80°C before the PLT plug was formed after PLT activation for subsequent determination of TGF-β1 and PDGF-BB concentrations.

### Hematological Analysis

Red blood cell (RBC; M/μL), white blood cell (WBC; K/μL), platelets (PLT; K/μL) and mean platelet volume (MPV; fL) count were determined in WB, PPP and PRP using an automated hematology analyzer (Advia® 2120i Siemens Healthcare Diagnostics Inc.). The absolute numbers of PLTs obtained by ADVIA were verified in blood smears always by the same clinical pathologist.

### Platelet-Derived Growth Factor-BB (PDGF-BB) and Transforming Growth Factor-β1 Quantification (TGF-β1)

TGF-β1 and PDGF-BB were measured after activation in both PPP and PRP fractions to assess the effect of the centrifugation in PLTs and associated GFs. TGF-β1 and PDGF-BB were measured using ELISA kits (Human TGF-beta 1 DuoSet ELISA de R&D Systems DY240-05 and Human PDGF-BB DuoSet ELISA de R&D Systems DY220, respectively) according to manufacturer's instructions. These GFs were determined using human antibodies because these kits have been used for the same purposes in other feline PC studies (20, 44) since it has been reported that human and cat PDGF-BB present high peptide sequence homology (45).

### Statistical Methods

The data were processed using the SPSS 20.0 program for Windows (SPSS® Inc., Chicago, USA).

A descriptive study of the mean, standard deviation and confidence intervals were made for each variable. A value of *p* < 0.05 was considered significant. Normality of data was tested in every quantitative variable with Shapiro-Wilk test and variance homogeneity with Levene test. ANOVA tests with a *post-hoc* Tukey test was used to compare normally distributed variables, while Non-parametric Kruskal-Wallis tests was used to compare the variables which did not follow a normal distribution. Correlations between variables were assessed using a Pearson correlation test.

## Results

Blood samples were drawn from a total of 30 healthy, neutered adult cats, 21 males and nine females. The mean age was 5.3 years old (range 2.7–7.9 years old) and the mean weight was 6.0 kg (range 4.87–7.13 kg).

Table 1 shows PLT concentration, MPV, number of RBC and WBC and the concentrations of PDGF-BB and TGF-ß1 in both PRP and PPP according to the three centrifugation protocols used in the study.

**Table 1:** Mean ± SD of platelet (PLT) concentrations, mean platelet volume (MPV), erythrocytes (RBCs) and leukocytes (WBCs) concentrations in whole blood samples (WB) and in the PRP and PPP fractions; platelet-derived growth factor BB (PDGF-BB) and transforming growth factor β1 (TGF-β1) concentrations in the PRP and PPP fractions in 30 cats (*n* = 30).

**Centrifugation protocol**		**255 g x 10 min**		**260 g x 10 min**		**265 g x 10 min**	
	**WB**	**PRP**	**PPP**	**PRP**	**PPP**	**PRP**	**PPP**
PLT (K/μL)	328.5 ± 155.8	462.1 ± 277.9	293.5 ± 179.1	426.0 ± 307.2	300.8 ± 180.7	481.4 ± 275.0	293.3 ± 161.4
MPV (fL)	16.3 ± 5.0	11.8 ± 1.9	11.25 ± 1.7	11.9 ± 2.6	11.3 ± 1.6	12.3 ± 2.5	11.4 ± 1.8
RBC (M/μL)	7.65 ± 1.23	0.07 ± 0.06	0.05 ± 0.056	0.07 ± 0.04	0.04 ± 0.02	0.07 ± 0.05	0.04 ± 0.021
WBC (K/μL)	9.46 ± 3.18	0.35 ± 0.45	0.17 ± 0.203	0.28 ± 0.26	0.15 ± 0.14	0.27 ± 0.28	0.12 ± 0.12
PDGF-BB (pg/ml)		1281.9 ± 1110.5	649.6 ± 596.8	1271.8 ± 965.7	559.9 ± 462.5	1346.6 ± 1197.8	616.6 ± 556.6
TGF-ß1(pg/ml)		17450.3 ± 10105.3	12441.1 ± 5165.9	14193.9 ± 7705.7	12499.1 ± 4962.9	17085.9 ± 7988.9	12404.1 ± 4539.6

### Platelet Concentration and Mean Platelet Volume

**Figure 2:**
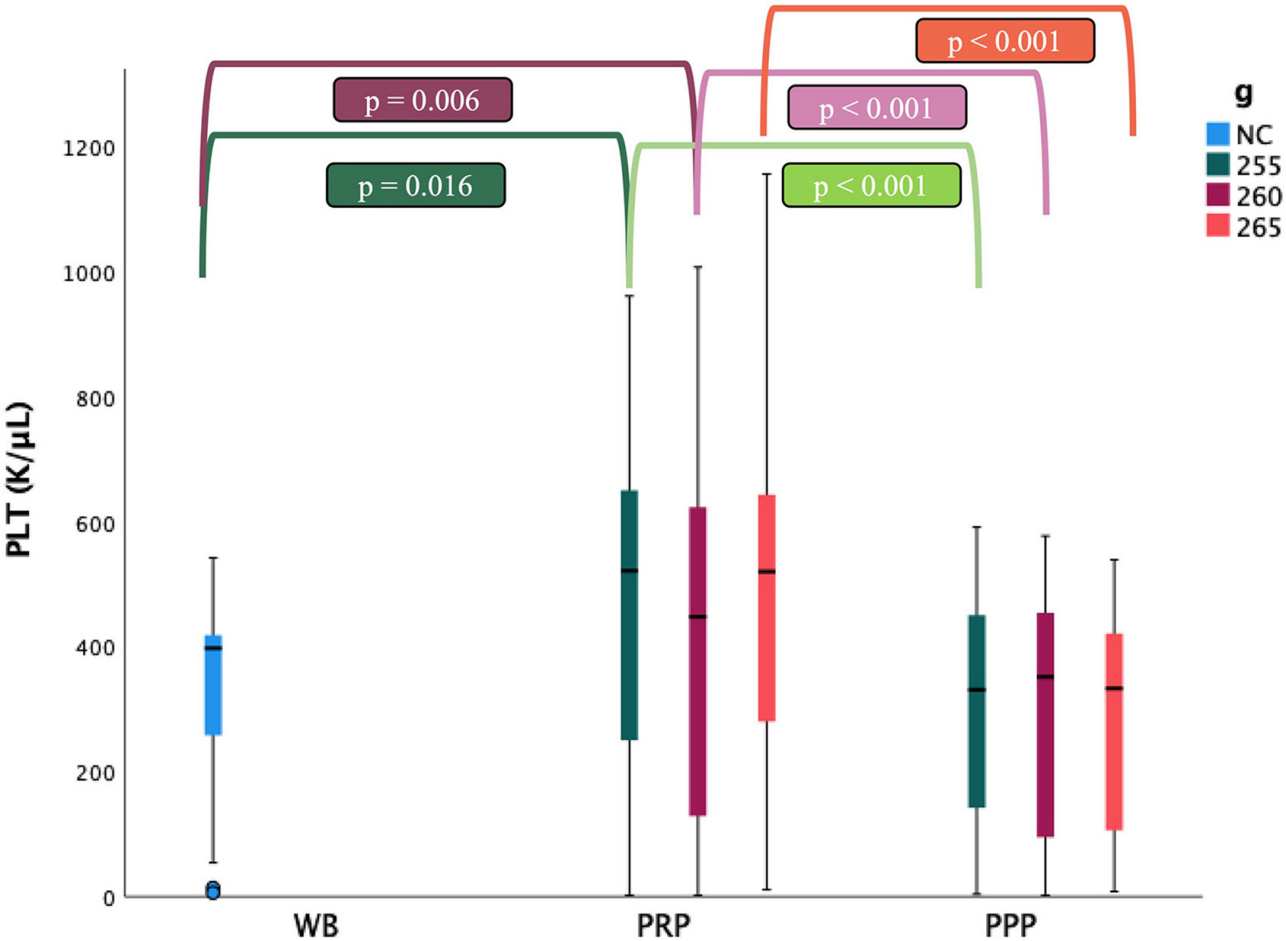
Comparison of the platelet (PLT) concentrations (mean ± SD) in cat (*n* = 30) between whole blood (WB), PRP and PPP fractions according to the three centrifugation protocols (g: 255, 260 and 265 in 10 min; NC: non-centrifugate). ∙: Outlyers data included in the statistically study.

The mean number of PLT was statistically higher in the PRP fraction than in WB (*p* = 0.026). The mean value of PLT in PRP fraction when centrifuging the samples at 255 g was 462.1 ± 277.9, 426.0 ± 307.2 K/ul at 260 g, and higher when using 265 g (481.4 ± 275.0 K/ul). In the PRP fraction, PLT concentration was increased 1.4 times on average at 255 g; 1.3 times on average at 260 g, and 1.5 times on average at 265 g. There were no statistical differences between the PPP and WB fraction. Using the centrifugation protocol at 255 g x 10 min, the number of PLT in the PRP fraction reached a mean value 1.5 times higher than the WB one in 14 (46.7%), 0.5 to 1.5 times in 10 (33.3%) and in <0.5 times in 6 (20%) of the samples analyzed. Compared with WB, the centrifugation protocol at 260 g x 10 min, the number of PLT in the PRP fraction increased more than 1.5 times in 11 (38%), from 0.5 to 1.5 times in 11 (38%) and in <0.5 times in 7 (24%) of the samples analyzed. By means of centrifugation protocol at 265 g x 10 min, the mean number of PLT increased more than 1.5 times in 13 (43.3%), from 0.5 to 1.5 in 14 (46.6%) and in <0.5 times in 3 (10%) of the samples analyzed. However, PLT concentrations did not differ significantly between the three protocols (Table 1; Figure 2).

A total of 206 samples were analyzed and PLT aggregates were obtained in 8 WB samples and 6 PRP samples. According to ADVIA 2120i (46) criteria, and the number of PLT aggregates in the smears, 13 out of the 14 samples, showed from 1 to 7 PLT aggregates with more than 10 PLTs each, while the remaining sample showed more than 50 PLTs almost in every PLT aggregate. Due to severe clustering after centrifugation, 2 PRP and 2 PPP samples were not included in the study.

The mean platelet volume (MPV) was statistically higher in WB than in PRP and PPP fractions (*p* < 0.001) without significant differences between protocols nor fractions (Table 1). MPV was positively correlated with the presence of PLT aggregates (*r* = 0.559, *p* < 0.001).

### Erythrocyte Concentration

Even though, the mean number of RBCs was statistically higher in WB than in the PRP and PPP fractions (*p* < 0.001), no significant differences were identified in relation to this parameter between both fractions. Compared to the initial value, the number of RBCs was reduced by 99% in all the analyzed samples, with no statistically significant differences between the three centrifugation protocols used in the study (Table 1).

### Leukocyte Concentration

The mean number of WBC was statistically higher in the WB samples than in the PRP and PPP fractions (*p* < 0.001), with no differences between them. Compared to the initial value, the number of WBC decreased more than 95% in the PRP and PPP fractions, without significant differences between protocols (Table 1).

### Platelet-Derived Growth Factor BB (PDGF-BB) Concentrations

**Figure 3:**
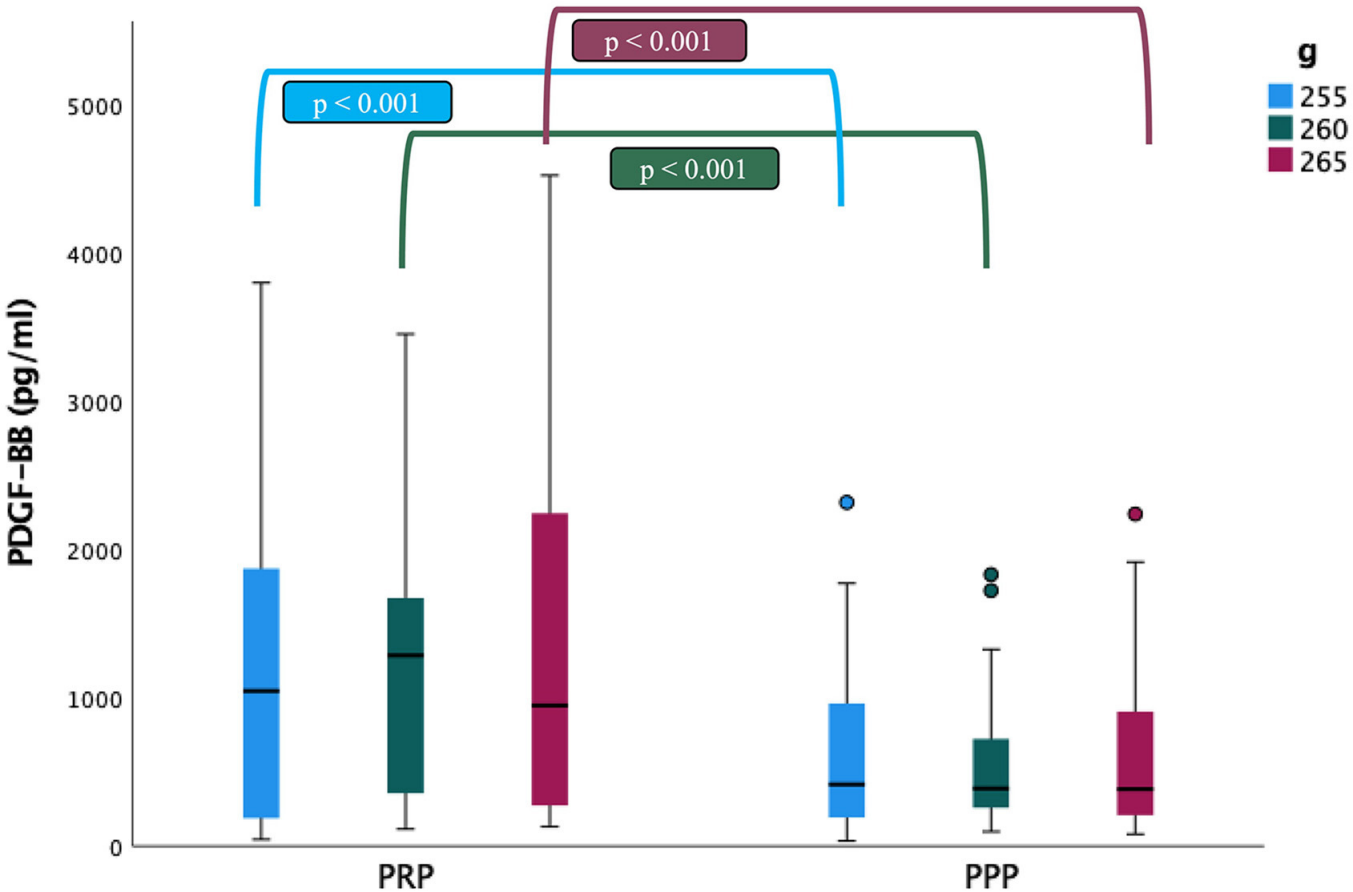
Comparison of the platelet-derived growth factor BB (PDGF-BB) concentrations (mean ± SD) in cat (*n* = 30) between PRP and PPP fractions according to the three centrifugation protocols (g: 255, 260 and 265 in 10 min). ∙: Outlyers data included in the statistically study.

The mean concentrations of PDGF-BB in the PRP fraction (1281.9 ± 1110.5, 1271.8 ± 965.7 and 1346.6 ± 1197.8 pg/ml) were statistically higher than those of the PPP fraction (649.6 ± 596.8 pg/ml, 559.9 ± 462.5 and 616.6 ± 556.6 pg/ml) (*p* < 0.001). The analysis of variation did not reveal statistically significant differences in the concentrations of PDGF-BB according to the centrifugation protocols used in the study (Table 1; Figure 3). The presence of PLT aggregates did not affect PDGF-BB concentrations in the PRP fractions.

### Transforming Growth Factor Beta 1 (TGF-ß1) Concentrations

**Figure 4:**
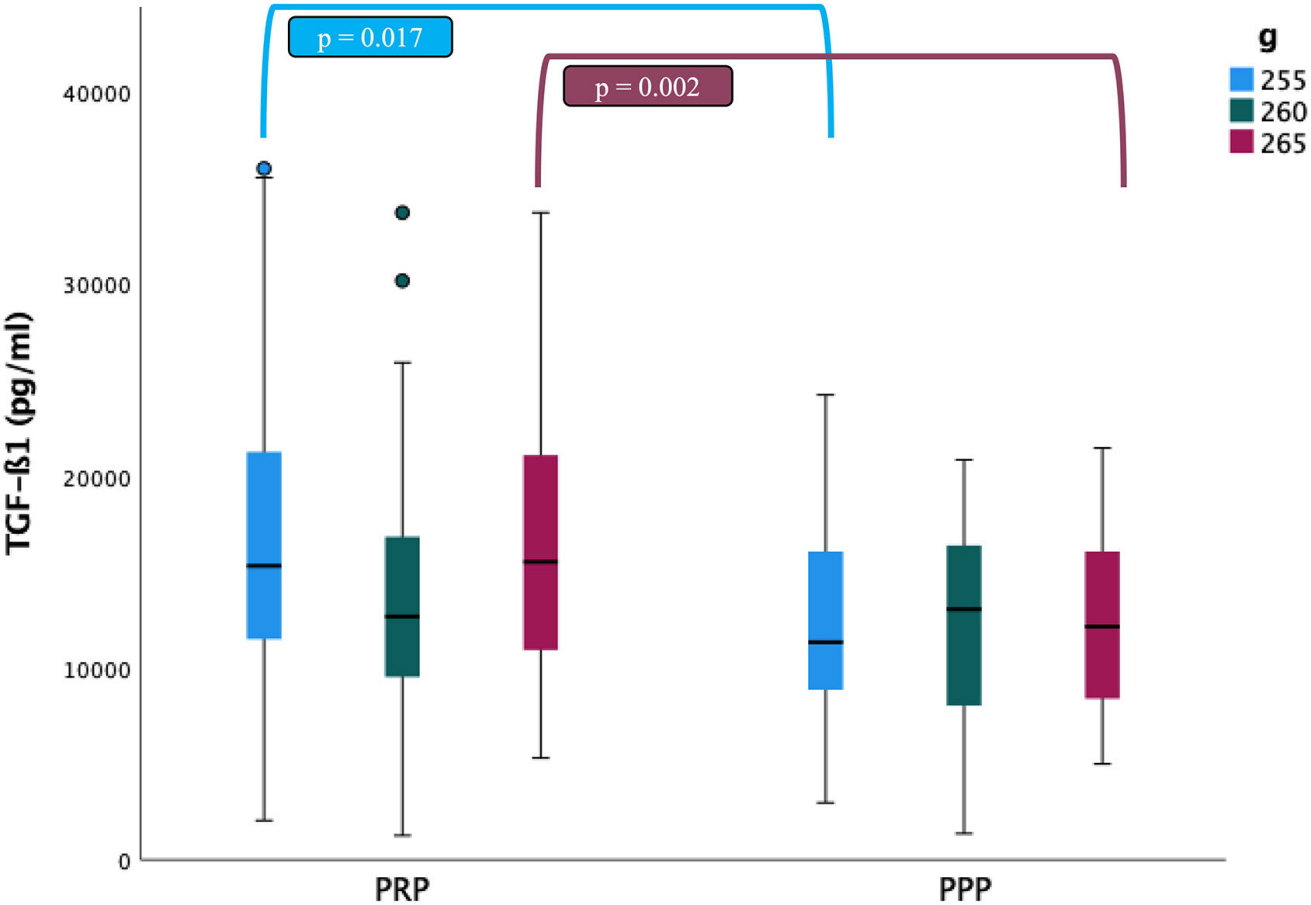
Comparison of the transforming growth factor β1 (TGF-ß1) concentrations (mean ± SD) in cat (*n* = 30) between PRP and PPP fractions according to the three centrifugation protocols (g: 255, 260 and 265 in 10 min). ∙: Outlyers data included in the statistically study.

The mean concentrations of TGF-ß1 in the PRP fraction (17450.3 ± 10105.3 and 17085.9 ± 7988.9 pg/ml) were statistically higher than those of PPP fraction (12441.1 ± 5165.9 and 12404.1 ± 4539.6 pg/ml) when centrifugation protocols at 255 g (*p* = 0.017) and 265 g (*p* = 0.002) were used. No significant differences were detected in TGF-ß1 concentrations among centrifugation protocols. The presence of aggregates also did not affect TGF-ß1 concentrations in the PRP fractions (Figure 4).

## Discussion

According to our hypothesis the mean PLT enrichment in the PRP fraction of the Endoret® preparations was 1.5 times higher (147%) than in WB at 265 g x 10 min, with proportions of 140% and 130% for 255 g x 10 min and 260 g x 10 min, respectively. In a similar way to what happens in other species such as rabbits and dogs (33, 34), the commercial system used in this study (PRGF®-Endoret®) increases the concentration of PLTs at least 1.5 times compared to baseline. The centrifugation protocols used in these studies yielded a mean PLTs concentration >300,000 plt/L, which is the minimum concentration required for a quality concentrate (47). However, some authors have described more moderate ranges of PLTs (1.5 to 2 times or 1.3 to 4 for WB) to provide greater clinical benefit in humans (48–51). It is documented that the protocols for PRP obtention show variations in the concentrations of PLTs, RBCs and WBCs among them for a specific species (52–54). In the same way, a validated protocol for a specific species may not produce the same cellular composition in any other species (22, 55). In other study, Boswell et al. affirmed that some individuals cannot concentrate platelets with a specific system, but contrarily, they are able to concentrate platelets successfully when using a system from a different manufacturer. This indicates that failure to generate PRP is possible in every individual and when using any system, therefore, it has been suggested that a complete blood count should be performed on each patient's venous blood and PRP, so the clinician can ensure that the patient is being treated with PRP (56). We have not obtained uniform results in all samples analyzed in our study due to individual characteristics and variability, as reported in previous studies.

The existing literature on PC in cats is scarce. In addition, there are no specific data on the exact range of PLTs required in a clot to be considered PRP in this species. In fact, although several commercial centrifugation methods showed increases in PLT between 151 and 187% (20–22) with respect to WB, they did not obtain PLT concentrations above the ideal range reported.

On the one hand, Silva et al. (20) collected WB samples cat in 8.5 mL tubes and after 6 min and 85 g centrifugation protocol obtained an 183% increase in PLT after spontaneous formation of the clot. Chun et al. (22) using WB volume of 12.5 mL, obtained PC by double centrifugation. After the first centrifugation at 3,600 rpm for 1 min, the plasma suspension was aspirated and centrifuged again at 3,800 rpm for 5 min, obtaining an increase in PLTs of 151%. On the other hand, Ferrari and Schwartz (21) compared two systems for obtaining PRP, using two volumes of 13.5 and 12.5 mL of WB. Although single centrifugation at 1,300 rpm for 5 min did not concentrate PLTs, double centrifugation at 3,600 rpm for 1 min and subsequently at 3,800 rpm for 5 min produced an increase in PLTs of 187% compared to the initial value (22, 55).

In our study, 9 mL tubes and a single centrifugation protocol were used, varying the force (255, 260, and 265 g) in a standard time of 10 min. To the author's knowledge, the WB sample volume, the gravitational force, and the centrifugation times considered in the different protocols for PRP obtention, could be responsible for the variations observed between studies, since the basal numbers of PLTs in WB were very similar. However, some characteristics such as the model or diameter of the centrifuge and the gravitational force were not specified in these studies, therefore, the authors emphasize the importance of characterizing the PRP product generated in a specific species by a given system.

PLT aggregation in feline blood samples is common, with reported prevalence ranging between 40% and 70% (21, 57–60). In this study, PLT aggregation occurred in 27% of the WB samples and in 7% of the PRP samples, with no evidence of PLT aggregation in the PPP samples. PLT aggregation is suspected to be due to species-specific factors, the sample collection method, and the anticoagulation protocol (21).

Several factors in the cat may be specifically involved in promoting aggregation tendency such as increased serotonin concentration, irreversible aggregation and release of granules when exposed to serotonin and irreversible aggregation in response to low concentrations of ADP (61). The quality of blood collection has been postulated to be the main cause of the presence of PLT aggregates in feline blood simples (62). The presence of PLT aggregates in these blood samples could be related to the collection method, since the WB samples were obtained directly by catheterization of the cephalic vein using a 22 G catheter, and it is well-known that vascular endothelial injury produced by venipuncture causes adherence of PLTs to von Willebrand factor bound to subendothelial collagen with PLT GPIbα receptors, inducing additional PLT recruitment (63).

On the other hand, baseline PLT counts in our study were performed using EDTA anticoagulated blood following the manufacturer's instructions. Although the nature of EDTA-induced pseudothrombocytopenia is still uncertain (64), it has been proposed that autoantibodies (IgG, IgM, and IgA) present in plasma recognize and bind to an epitope of glycoprotein IIb (GPIIb), which is part of the PLT surface GPIIb/IIIa complex, promoting PLT agglutination (65). PLT counts by ADVIA were reduced in samples with EDTA-induced pseudothrombocytopenia, whereas PLT aggregates were observed in blood smears. In fact, in 7 samples, from 1 to 7 groups with> 10 PLTs were identified and in 1 sample most of the cells were forming clusters of > 50 PLTs. This reduction in the number of baseline PLT in the samples with pseudothrombocytopenia using ADVIA could explain the wide range of variation experienced in our results since, like the rest of the samples, they were also included in the statistical study. Although the presence of pseudothrombocytopenia in our study was low, based on previous evidence, it seems that EDTA is not the appropriate anticoagulant for feline blood.

Currently, antiaggregants such as prostaglandin E1 (PGE1) and prostaglandin I2 (PGI2) (66) are used to prevent PLT aggregation in cats. Iloprost is a PGI2 analogous that inhibits the reactions induced by aggregating agents such as arachidonic acid, collagen or epinephrine (62). Since Iloprost does not influence cytological evaluations of blood smears or other hematological parameters, the addition of these substances to EDTA tubes is a good alternative to avoid PLT aggregation and increase the reliability of feline PLT counts.

Compared with previous results (20) in which MPV was lower in WB than in PC, MPV in WB in this study was significantly higher than in both PRP and PPP fractions regardless of the centrifugation protocol used. Certain factors such as the type of anticoagulant used, the time required to perform the analysis, and the methodology (67) could be some of the factors involved in these differences. Indeed, the MPV greatly depends on the measurement technique and on the duration and conditions of storage before the blood is tested. ADVIA is considered to have one of the best methods for the analysis of these cells as it identifies size, optical density and detects PLTs up to 60 fL (57). Feline blood can contain PLTs larger than 60 fL (66), so ADVIA may not detect them. This failure to detect larger PLTs may be the reason why the number of PLTs by ADVIA is falsely lower in some of the samples tested. The increases in MPV correlated with the decrease in the number of PLTs could indicate that these blood samples had larger PLTs (66). Regarding the type of anticoagulant, PLTs swell when stored in EDTA and, to a lesser extent, in solutions containing citrate, making the determination of MPV dependent on time (66).

Our study reported lower serum TGF-ß1 values than those previously reported by Arata et al. (44). However, both the serum concentrations of TGF-ß1 and those of PDGF-BB were higher in the present study than the concentration obtained in plasma by Silva et al. (20). Several factors such as the base fluid (plasma or serum), the sample volume and the PLT activation method used could have conditioned the differences between the results obtained in the latest research (55). Serum differs from plasma in that most of the fibrinogen is converted into a fibrin clot in which PLTs can be attached to the fibrin matrix, activated to form aggregates or both (68). Based on these events, it has been postulated that PLTs in serum samples undergo premature activation compared to plasma samples, with the consequent release of GFs contained in the α-granules of PLTs (20). The concentration of PDGF was higher in PRP than in PPP fraction with all centrifugation protocols. However, TGF-ß1 was more concentrated in PRP than in PPP fraction when centrifugation protocols at 255 and 265 g were used. Further studies are needed to evaluate the temporal release kinetics after PLT activation and the distribution of GFs in PLT α-granules in cats. Conversely, a major finding of this study is an increased of PDGF-BB and TGF-ß1 in samples with PLT clumping with no significant differences to TGF-ß1. This suggests that pseudothrombocytopenia in cats does not affect the concentration of GFs such as PDGF-BB and TGF-ß1. Nevertheless, studies on PRP in cats are scarce, and further investigation is required to determine the optimal concentrations of GFs in PRP products in cats to achieve clinical efficacy in cats.

PRP has been of interest in the field of regenerative medicine due to the regenerative potential of its GFs (1). However, there is considerable variability in the platelets' gathering and the concentration of GFs within different PRP preparations. There are many PRP-purification systems commercially available (69), and prepared PRP varies in terms of PLT concentration, degree of WBC, RBC, and concentration of GFs (50). PRGF®-Endoret® technology is a type of PRP obtained from small volumes of blood through a single centrifugation process and subsequent fractionation of the plasma. This is important in cat practice because the volume of blood required to obtain PRP for clinical use could be a limiting factor due to the small size of this species. The volume of blood extracted in our study did not exceed the maximum volume of extraction allowed (21, 70) in any cat, thus guaranteeing correct volemia and perfusion. In addition, PRGF®-Endoret® technology is classified as P-PRP, is moderately enriched in PLTs, does not contain RBCs or WBCs, and its activation is performed with calcium chloride (29). These criteria are met in the 3 protocols used in our study, in which the presence of RBCs and WBCs was reduced to values close to zero. Similar results were shown by Ferrari and Schwartz (21) using a simple centrifugation system (1,300 rpm x 5 min), however, when they used a double centrifugation system (3,600 rpm × 1 min and 3,800 rpm × 5 min), a 80% reduction in WBCs was reported, resulting in a PRP product characterized by the presence of WBCs. There are different PRP formulations according to the presence or absence of WBC: Leukocyte poor PRP (LP-PRP), P-PRP and LR-PRP (24, 71, 72). Taking this classification into account, the double centrifugation protocol used by Ferrari and Schwartz (21) provided a LP-PRP. Nevertheless, the presence or absence of WBCs in PRP is frequently discussed in the literature. WBC could be an additional source of GFs and antimicrobial properties (54, 73) but, in the other hand, WBC release proinflammatory cytokines that could prompt the inflammatory process and counteract the positive effect on tissue regeneration (54, 71). Our study did not assess the clinical efficacy of PRGF products obtained, and it is unknown if these products with moderate PLT concentration and undetectable RBC and WBC would have applications *in vivo*. The versatility and biocompatibility of these products have stimulated their therapeutic use in numerous medical and scientific fields including dentistry, oral implantology, orthopedics, ulcer treatment, and tissue engineering among others. The use of PRGF in the treatment of osteoarthritis in dogs and rabbits (8, 10, 74), tendinopathies in horses (75–77) and sheep (78), wound healing in rabbits (9, 79) or corneal ulcer healing in different species (80, 81) is promising. Cats are susceptible to various musculoskeletal diseases and can also suffer traumatic damage (82), in addition, in recent years more orthopedic diseases have been identified in cats, especially osteoarthrosis (22). It is reasonable to assume that PRGF therapy could be effective for feline patients in a similar way to what occurs in other animal species for the treatment of osteoarthritis, wound healing, and tendinopathies (21).

Future research is needed to evaluate the clinical efficacy of PRGF obtained by PRGF®-Endoret® technology in this species. The standardization and optimization of the PGRF obtained by means of this methodology would allow to formulate the preparations according to each specific pathology (29).

## Conclusions

The BTI-Endoret® methodology allows to obtain unnoticeable RBC and WBC in the PRP fractions of healthy cats, so it could be considered P-PRP and centrifugation at 265 g for 10 min shows a mean PLT enrichment 1.5 times higher in Endoret® preparations than baseline. Moreover, even if no statistical differences have been observed between different protocols, when using 265 g for 10 min a higher PLT concentration and lower concentration of WBC were obtained. Following PRGF®-Endoret® technology greater values of PDGF-BB and TGF-ß1 than in basal serum samples have been obtained in previous studies. Further studies are needed to determine the technique reproducibility and validation and to evaluate safety and benefit degree for feline patients.

## Data Availability Statement

The raw data supporting the conclusions of this article will be made available by the authors, without undue reservation.

## Ethics Statement

The animal study was reviewed and approved by CEU Cardenal Herrera University Committee of Ethics in Animal Research (code: 2018/VSC/PEA/0196). Written informed consent was obtained from the owners for the participation of their animals in this study.

## Author Contributions

LM-P performed the experiment, analyzed data, and wrote the manuscript. DC, MT-T, AR, PP, and BC participated in performing the experiment. LM-P and KS developed the first draft of the manuscript. JC, MR, and JS designed the study, supervised all procedures, and coordinated the research. MR and KS performed statistical analysis. JC analyzed blood samples. JS proofread the manuscript and gave final approval of the version. All authors have read and agreed to the published version of the manuscript.

## Funding

This research was funded by García Cugat Foundation CEU-UCH Chair of Medicine and Regenerative Surgery and the project INDI21/54 CEU-UCH.

## Conflict of Interest

The authors declare that the research was conducted in the absence of any commercial or financial relationships that could be construed as a potential conflict of interest.

## Publisher's Note

All claims expressed in this article are solely those of the authors and do not necessarily represent those of their affiliated organizations, or those of the publisher, the editors and the reviewers. Any product that may be evaluated in this article, or claim that may be made by its manufacturer, is not guaranteed or endorsed by the publisher.
