# Corrigendum: Evaluation of a Standardized Protocol for Plasma Rich in Growth Factors Obtention in Cats: A Prospective Study

**DOI:** 10.3389/fvets.2022.954516

**Published:** 2022-06-17

**Authors:** Laura Miguel-Pastor, Katy Satué, Deborah Chicharro, Marta Torres-Torrillas, Ayla del Romero, Pau Peláez, José M. Carrillo, Belén Cuervo, Joaquín J. Sopena, José J. Cerón, Mónica Rubio

**Affiliations:** ^1^Bioregenerative Medicine and Applied Surgery Research Group, Department of Animal Medicine and Surgery, CEU Cardenal Herrera University, CEU Universities, Valencia, Spain; ^2^García Cugat Foundation CEU-UCH Chair of Medicine and Regenerative Surgery, CEU Cardenal Herrera University, CEU Universities, Valencia, Spain; ^3^Interdisciplinary Laboratory of Clinical Analysis, University of Murcia, Murcia, Spain

**Keywords:** cat, platelet count, Platelet-Rich Plasma, platelet-derived growth factor BB, transforming growth factor β1, PRGF®

In the original article, there was an error in the Conclusions section, paragraph 1: “when using 250 g for 10 min a higher PLT concentration and lower concentration of WBC were obtained.” The correct text is “when using 265 g for 10 min a higher PLT concentration and lower concentration of WBC were obtained.”

The authors apologize for this error and state that this does not change the scientific conclusions of the article in any way. The original article has been updated.

## Publisher's Note

All claims expressed in this article are solely those of the authors and do not necessarily represent those of their affiliated organizations, or those of the publisher, the editors and the reviewers. Any product that may be evaluated in this article, or claim that may be made by its manufacturer, is not guaranteed or endorsed by the publisher.

